# An Efficient Genome-Wide Fusion Partner Screening System for Secretion of Recombinant Proteins in Yeast

**DOI:** 10.1038/srep12229

**Published:** 2015-07-21

**Authors:** Jung-Hoon Bae, Bong Hyun Sung, Hyun-Jin Kim, Soon-Ho Park, Kwang-Mook Lim, Mi-Jin Kim, Cho-Ryong Lee, Jung-Hoon Sohn

**Affiliations:** 1 Bioenergy and Biochemical Research Center, Korea Research Institute of Bioscience and Biotechnology (KRIBB), Daejeon 305-806, Republic of Korea; 2 Biosystems and Bioengineering Program, University of Science and Technology (UST), Daejeon 305-350, Republic of Korea

## Abstract

To produce rarely secreted recombinant proteins in the yeast *Saccharomyces cerevisiae*, we developed a novel genome-wide optimal translational fusion partner (TFP) screening system that involves recruitment of an optimal secretion signal and fusion partner. A TFP library was constructed from a genomic and truncated cDNA library by using the invertase-based signal sequence trap technique. The efficiency of the system was demonstrated using two rarely secreted proteins, human interleukin (hIL)-2 and hIL-32. Optimal TFPs for secretion of hIL-2 and hIL-32 were easily selected, yielding secretion of these proteins up to hundreds of mg/L. Moreover, numerous uncovered yeast secretion signals and fusion partners were identified, leading to efficient secretion of various recombinant proteins. Selected TFPs were found to be useful for the hypersecretion of other recombinant proteins at yields of up to several g/L. This screening technique could provide new methods for the production of various types of difficult-to-express proteins.

Recombinant production of valuable proteins that are expressed in limited amounts in nature is essential for generation of human biopharmaceuticals and industrial biocatalysts. Therefore, it is necessary to develop a stable, easy-to-use system for high-level expression and large-scale purification of useful proteins. In this regard, the eukaryotic yeast *Saccharomyces cerevisiae* has been generally recognised as safe (GRAS) for humans and has advantages over other expression systems, such as *Escherichia coli* and mammalian cells. *S. cerevisiae* has a secretion pathway and post-translational modification system, similar to those of higher eukaryotes, except the possibility of hyperglycosylation of the recombinant proteins. Thus, it can produce various complex proteins originating from higher eukaryotes at low cost, which are often expressed in inactive forms as inclusion bodies in *E. coli*.

Although yeast has such advantages for producing foreign proteins, the productivity of the system can be unpredictable. Yeasts have the capacity to secrete several grams of protein per litre of culture. However, output varies greatly according to the protein type. For example, human serum albumin (HSA) and human gelatin can be secreted at up to several g/L in *Pichia pastoris*^1^,^2^. In contrast, the secretory productivities of human pharmaceutical proteins such as human interleukin (hIL)-2, granulocyte-colony stimulating factor (GCSF), and platelet-derived growth factor (PDGF) are extremely low even in *P. pastoris*^3^,^4^,^5^. Due to such discrepancies among proteins, the secretory productivity of a foreign protein cannot be predicted until it is measured. Such limitations must be overcome to develop a general secretion system for recombinant proteins.

Numerous studies have sought to improve our understanding of the secretory pathway of proteins and to enhance the secretion of rarely secreted proteins in yeast^6^. Overexpression of a foreign protein through the secretory pathway can lead to saturation of the folding machinery, typically in the endoplasmic reticulum (ER)^7^ . The secretion rate of overexpressed proteins is mainly dependent on the folding rate of the proteins in the ER^8^. Unless the proteins fold correctly, they cannot pass through the secretory pathway due to the ER quality control system^9^. Misfolded proteins are accumulated in the ER as aggregates, triggering the unfolded protein response, and are eventually degraded by the ER-associated degradation pathway^10^. Two different approaches, *trans*-acting chaperones^11^,^12^ and *cis*-acting chaperones (secretory fusion partners)^13^,^14^,^15^, have been used to enhance protein folding rates and avoid aggregation and degradation. Although such *trans*- and *cis*-acting chaperones have been used to improve the secretion efficiency of some proteins, many negative examples, often unpublished, have also been reported for other proteins with the same chaperones^16^,^17^,^18^. Engineering of secretion signal peptides by rational design or directed evolution has increased protein secretion in some cases^19^,^20^. However, these synthetic secretory peptides do not function well for all heterologous proteins. These results suggest that one particular chaperone cannot play an omnipotent function for facilitating the secretion of every protein. Thus, it is necessary to identify protein-specific chaperone(s) to enhance target protein secretion.

In this study, we describe a novel approach for the selection of a target protein-specific secretion fusion partner from a genome-wide library constructed by the invertase-based secretion signal trap technique^21^,^22^,^23^. Direct fusion of a rarely secretable target protein to the N-terminus of invertase did not support cell growth on sucrose media because of the secretion-blocking effect of the target protein on invertase. Although the reason for the secretion block may be variable according to the target protein, it is conceivable that the insertion of a proper secretion signal and fusion partner in front of such problematic fusion proteins could support cell growth on sucrose media. Consequently, high-throughput, positive selection of a target protein-specific translational fusion partner (TFP) harbouring a secretion signal and fusion partner from truncated genomic and cDNA libraries could be performed by simple selection of cells growing on sucrose medium. This method could be used to identify various secretion signals and fusion partners from the TFP library for the secretion of individual proteins.

## Results

### Trapping of optimal TFPs from a genomic DNA library for secretion of hIL-2, a rarely secreted protein in yeast

**Figure 1:**
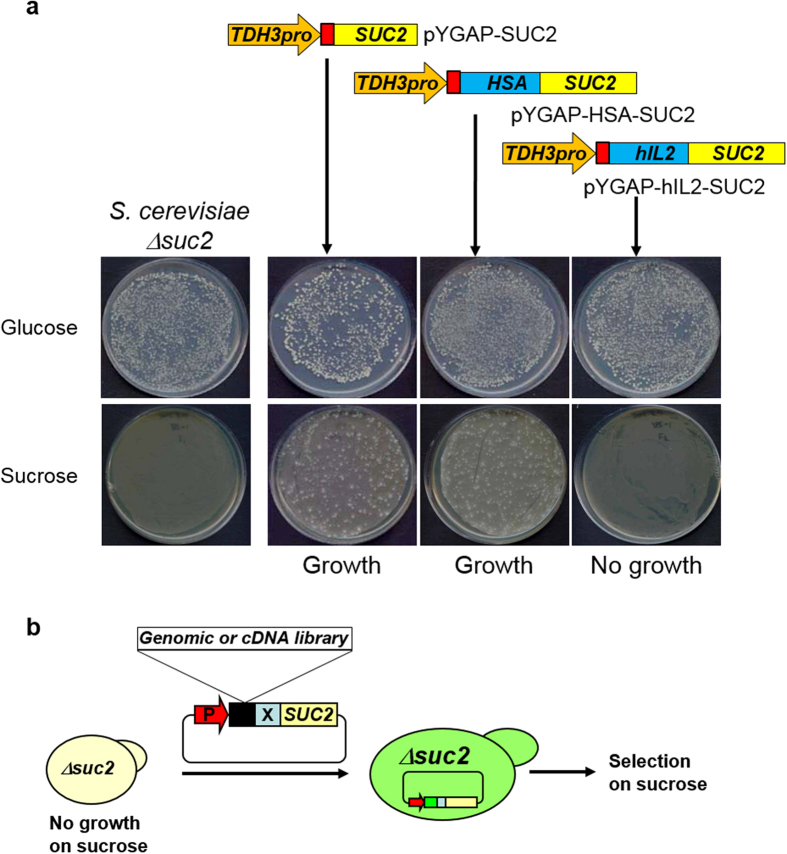
Invertase-based translational fusion partner trap system. (**a**) Growth patterns of *Saccharomyces cerevisiae Y2805 Δsuc2* recombinant strains carrying the indicated plasmids on different carbon sources, glucose and sucrose. (**b**) Schematic representation of the genetic screening system for the cloning of optimal translational fusion partners (TFPs) to accelerate secretion of target proteins (X).

Secretion of invertase, encoded by *SUC2*, is prerequisite for yeast cell growth on media containing sucrose as a sole carbon source. The growth defect of the invertase deletion mutant *S. cerevisiae* Y2805Δ*gal80*Δ*suc2* on sucrose medium was recovered by functional complementation of the *SUC2* gene (Fig. 1a). Interestingly, we found that invertase exhibited different fates when it was directly fused to HSA or hIL-2. The expression of the HSA-SUC2 fusion protein supported growth of the invertase mutant strain on sucrose medium, whereas the expression of hIL2-SUC2 did not support growth on sucrose medium. Therefore, fusion of the well-secreted HSA protein did not disturb invertase secretion. On the other hand, fusion of the rarely secreted hIL-2 protein completely blocked the secretion of invertase. This feature of invertase fused with a low-secretion-competent protein (X) like hIL-2 made it possible to design a genetic trap for target protein-specific TFPs from a fusion partner library derived from truncated cDNAs and genomic DNA (Fig. 1b).

To identify an optimal TFP for the efficient secretion of hIL-2, partially digested genomic DNA of *S. cerevisiae* was integrated in front of *hIL2-SUC2* and introduced into Y2805Δ*gal80*Δ*suc2* (Fig. 1b). Eleven transformants were formed on sucrose medium. Sequence analyses of the plasmids from the respective colonies revealed the presence of three different TFPs (i.e., TFP1, −2, and −3). The sizes of the TFPs varied from 103 to 117 amino acids (aa). All TFPs originated from known open reading frames (ORFs) encoding secretory proteins with pre- or prepro-secretion signal sequences and stretches of fusion peptides (Table 1). YAR066w (TFP1) is a hypothetical ORF containing 23 aa of a putative signal sequence and a glycosylphosphatidyl inositol (GPI)-anchor motif. *ULI1* (TFP2) encodes a putative protein of unknown function induced by the ER unfolded protein response and contains 19 aa of a putative signal sequence. *CIS3* (TFP3), a *cik1* suppressor with internal repeats, encodes a cell wall protein with 21 aa of a putative pre-signal and 43 aa of a pro-sequence processed by Kex2p^24^.

### Identification of an essential domain of TFP1 for protein secretion

**Figure 2:**
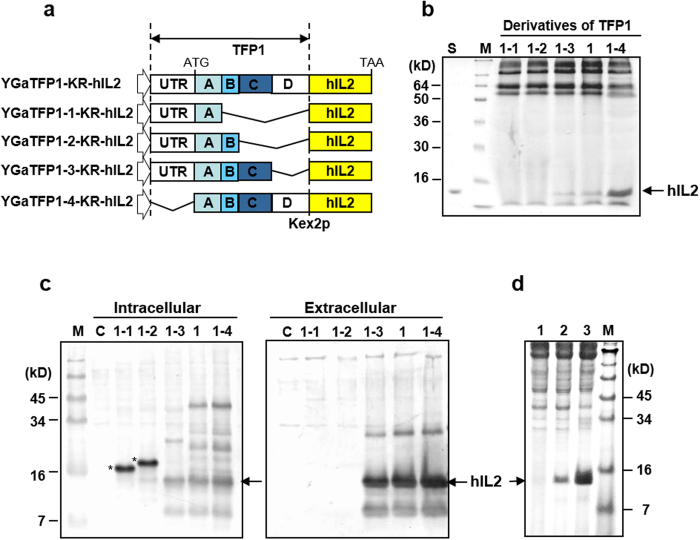
Effects of the translational fusion partner (TFP) 1 domains on the secretion of a rarely secreted protein, human interleukin (hIL)-2. (**a**) Schematic diagram of TFP1 derivatives. TFP1 was divided into five domains (UTR: 5′ untranslated region, A: signal sequence, B: N-glycosylation site, C: Ser and Ala rich sequence, D: a flanking sequence). Deleted domains indicated by the bent line. (**b**) SDS-PAGE analysis of hIL-2 secreted into the culture medium by recombinant strains carrying the plasmids harbouring TFP1 derivatives. Lane S: recombinant hIL-2 produced by *Escherichia coli.* M: standard protein size marker. The protein is revealed by Coomassie staining. (**c**) Western blot analysis of intracellular and extracellular proteins produced by recombinant strains carrying the indicated plasmids. C: host strain carrying the mock vector. (**d**) SDS-PAGE followed by Coomassie staining for the comparison of hIL-2 secretion using different signal sequences. Lane 1: α-amylase signal peptide from *B. subtilis,* lane 2: prepro signal peptide of mating factor α from *S. cerevisiae*, and lane 3: TFP1-4 in this study.

To ensure secretion of hIL-2 using the screened TFPs without invertase, the *SUC2* gene of each vector was removed, and the yeast dipeptidyl protease Kex2p^25^ cleavage site (LDKR) was inserted between *TFP* and *hIL2* by PCR. Among three tested TFPs, TFP1 was superior to the other TFPs for hIL-2 secretion (data not shown). The TFP1 nucleotide sequence contained five domains that might be important for secretion of hIL-2 (domain A: signal sequence, B: N-glycosylation site, C: Ser- and Ala-rich sequence, D: a flanking sequence, and a 5′-untranslated region [UTR]). Four truncated TFP1 derivatives were constructed to identify the effects of each domain on hIL-2 secretion (Fig. 2a), and the culture supernatants of each construct were analysed by SDS-PAGE (Fig. 2b). The hIL-2 protein fused with TFP1-1 and TFP1-2, containing the signal sequence or N-glycosylation site only, was not secreted or even detected by western blot analysis, but was detected in the intracellular fraction of each cell (Fig. 2c). The slightly larger size of these proteins compared with the secreted hIL-2 indicated that they were not processed by Kex2p. Therefore, these fusion proteins may not reach the Golgi complex and would be caught by the ER quality control system. This result clearly showed that hIL-2 was not secreted by a traditional approach employing only a secretion signal. TFP1-3, containing domains A, B, and C, secreted authentic hIL-2 with a small amount of unprocessed and degraded protein, but still less than that of the original TFP1 (Fig. 2b), suggesting that all TFP1 domains were necessary for hIL-2 secretion. Finally, hIL-2 secretion was augmented by about three-fold after removing the 5′-UTR (TFP1–4) (Fig. 2b). Some yeast genes are known to contain negative *cis*-acting elements that affect the translational efficiency on the 5′-UTR^26^. The secretion-enhancing effects of TFP1-4 were compared with other well-known signal sequences often used for secretion of recombinant proteins in yeast. Compared with the α-amylase (AMY) signal peptide from *Bacillus subtilis* and mating factor α signal peptide (MFα) of *S. cerevisiae*, TFP1-4 dramatically improved hIL2 secretory productivity by about 10-fold compared with that of MFα and several hundred-fold compared with that of AMY, respectively (Fig. 2d).

**Figure 3:**
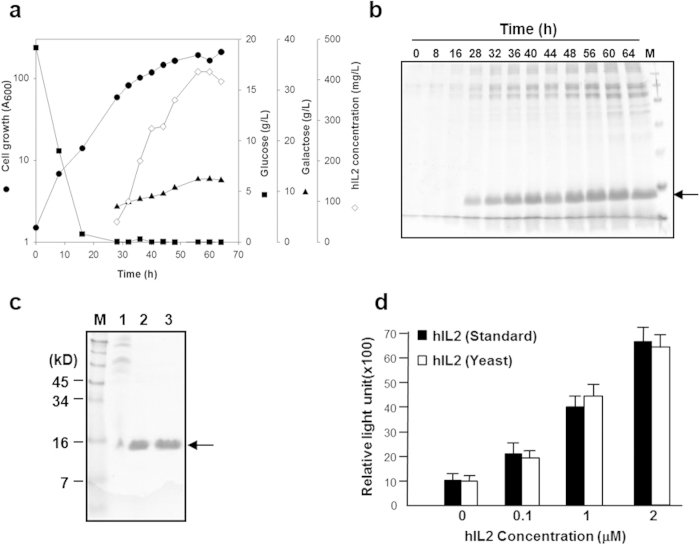
Production and characterization of hIL-2 directed by translational fusion partner (TFP) 1–4 in *S. cerevisiae.* (**a**) Profile of fed-batch fermentation. Closed circles: cell growth, closed square: concentration of glucose, closed triangle: concentration of galactose, open diamond: concentration of hIL-2. (**b**) SDS-PAGE analysis of 10-μL aliquots of fermentation broth retrieved at the indicated times. M: size markers. (**c**) SDS-PAGE for the purified hIL-2. Lane 1: after ultrafiltration, lane 2: after ion-exchange chromatography, and lane 3: after gel filtration chromatography. (**d**) Bioactivity assay of the purified hIL-2. The EL-4 cell line was cultured in the presence of indicated amount of hIL-2, and cell proliferation was analysed by bromodeoxyuridine (BrdU) labelling. The protein was revealed by Coomassie staining.

Fed-batch fermentation of the Y2805 strain harbouring YGaTFP1-4-hIL2 was carried out to confirm the productivity of hIL-2. The yield of secreted hIL-2 was over 400 mg/L from 40 g DCW/L (Fig. 3a,b), as determined by a density comparison with standard hIL-2 produced by *E. coli*. The intactness of the secreted recombinant hIL-2 was confirmed by N-terminal amino acid sequencing and mass analysis (data not shown). To analyse the activity of secreted recombinant hIL-2, the protein was purified by ion-exchange and gel-filtration column chromatography (Fig. 3c). The biological activity of the purified hIL-2 was determined by an *in vitro* proliferation assay using the EL-4 (mouse T-lymphocyte) cell line (Fig. 3d). Recombinant yeast hIL-2 showed biological activity similar to that of standard hIL-2, suggesting that the hypersecreted yeast hIL-2 produced using the TFP technology developed in this study was fully intact. Furthermore, this screening system could directly retrieve optimal TFPs facilitating the hypersecretion of low-secretion-competent proteins.

### High-throughput screening of TFPs

**Figure 4:**
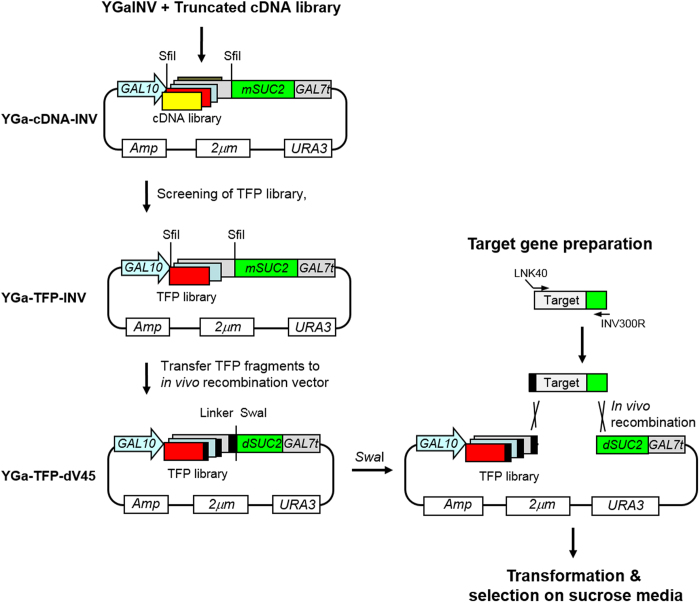
Schematic process for high-throughput screening of translational fusion partners (TFPs) for various target proteins. The 3′-end truncated cDNA library was constructed in YGaINV vector containing mature invertase gene (*mSUC*2). TFPs accelerating secretion of invertase were collected from a cDNA library and transferred to an *in vivo* recombination vector, containing a linker (black bar) and a defective invertase gene (*dSUC2*) to construct a TFP library, YGa-TFP-dV45. Target genes were flanked with the linker and N-terminal invertase fragments by overlap extension PCR and transformed with the *Swa*I linearised TFP library.

Although the TFP trap system is powerful for the selection of an optimal TFP for a target protein, high-throughput application of this technology for many target proteins is restricted by the requirement for recursive construction of a library. To solve this problem and further confirm the TFP trap technology, we constructed a TFP library that could be easily applicable to a large number of target proteins. About 3 × 10^3^ TFPs secreting invertase were collected from the 3′-end truncated cDNA library in YGa-TFP-INV, which contained TFPs for mature invertase (*mSUC2*) secretion (Fig. 4). To reduce the background level of transformants on sucrose media, *Sfi*I-digested TFP fragments of YGa-TFP-INV were subcloned into YGa-TFP-dV45 containing a defective *SUC2* (d*SUC2*) in which the N-terminal 45 aa were deleted (Fig. 4). Test transformation of the YGa-TFP-dV45 library into invertase-deficient *S. cerevisiae* produced thousands of transformants on UD medium, but no transformants on sucrose medium. Functional invertase could be recovered only when the *Swa*I-linearised YGa-TFP-dV45 plasmid containing the TFP library and a target gene flanked with the linker and N-terminal parts of invertase were co-transformed into yeast, and the circular plasmids were reconstructed by *in vivo* recombination (Fig. 4). The *hIL2* gene was used again as a model target protein to screen for optimal TFPs. Overlap extension PCR was performed to flank the *hIL2* gene with linker DNA and a 0.3-kb DNA fragment encoding the N-terminal part of invertase at the 5′ and 3′ ends, respectively. Then, the *Swa*I-digested YGa-TFP-dV45 plasmid containing the TFP library and the insert fragment were co-transformed into invertase-deficient *S. cerevisiae* Y2805*Δgal80Δsuc2.* Correct insertion of the target gene between an optimal TFP rendering secretion of hIL-2 and d*SUC2* by *in vivo* recombination supported the growth of transformants on sucrose medium. Approximately, 2 × 10^4^ transformants were formed on UD medium, whereas 24 transformants were obtained on sucrose medium.

**Figure 5:**
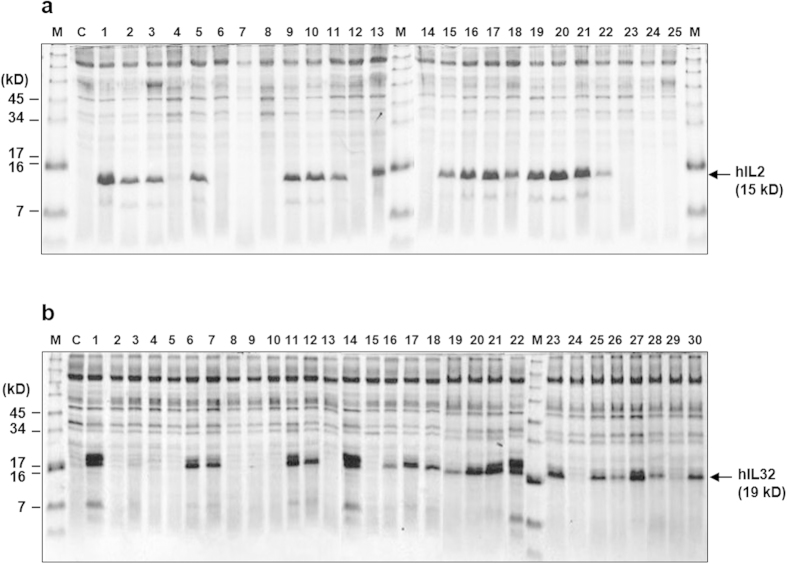
SDS-PAGE analysis of hIL-2 (a) and hIL-32 (b) expressed by randomly selected translational fusion partners from TFP library. A 0.6-mL aliquot of culture supernatant was precipitated with acetone and analysed on a 10% Tricine gel. M: standard protein size marker, C: host strain carrying a mock vector, (**a**) Lane 1: TFP1-4, lane 2, 9, 13: TFP5, lane 3: TFP18, lane 5, 22: TFP19-1, lane 10: TFP16-1, lane 11, 15, 18: TFP17-1, lane 16: TFP5-3, lane 17, 19, 20, 21: TFP17-3. (**b**) Lane 1, 14, 22: TFP10, lane 6, 11, 21, 23: TFP5-1, lane 7: TFP6, lane 12: TFP7-1, lane 16: TFP18-1, lane 17: TFP20, lane 18, 25: TFP5-2, lane 19: TFP11, lane 20: TFP14-1, lane 26: TFP21, lane 27: TFP16-3, lane 28: TFP21, lane 30: TFP22. The protein was revealed by Coomassie staining.

To confirm actual hIL-2 secretion, plasmids were recovered from 24 transformants, and hIL-2 expression vectors were reconstructed to express *hIL2* without *SUC2* directed by respective TFPs. Around 60% of TFPs secreted hIL-2 into culture supernatants with variable efficiencies (Fig. 5a). Several TFPs showed efficiencies comparable to that of TFP1–4, but some TFPs secreted only small amounts of hIL-2, which were detectable by western blotting with an anti-hIL-2 antibody (data not shown). Nucleotide sequencing of TFPs secreting hIL-2 revealed seven different TFPs. To obtain additional TFPs, we repeated this process two times more and finally obtained 28 different TFPs, as summarised in Table 1. All TFPs originated from various types of secretory proteins, such as cell wall proteins, plasma and vacuolar membrane proteins, and functionally unknown secretory proteins. Some proteins were repeatedly found with different sizes from the same genes (TFP3, −5, −11, −16, −17, and −19). Indeed, most of the yeast proteins passing through the ER could be TFP candidates. Consequently, there were numerous unidentified TFP candidates in the yeast genome that could improve the secretion competency of hIL-2. Among them, TFP1–4 seemed to be an optimal TFP for secretion of hIL-2.

**Figure 6:**
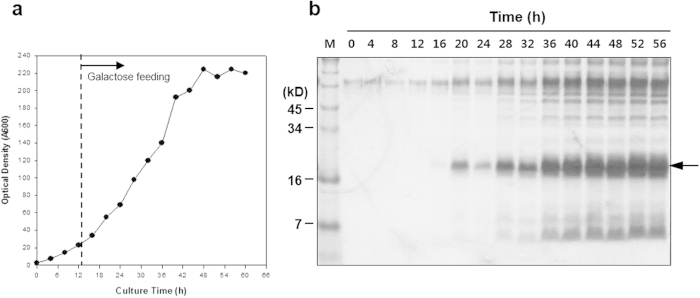
Fermentation of recombinant yeast secreting hIL-32 directed by TFP10. (**a**) Profile of fed-batch fermentation, (**b**) SDS-PAGE followed by Coomassie staining analysis of fermentation broth (10 μL) retrieved at the indicated times, lane M: standard protein size marker.

To confirm the TFP trap technology using another rarely secreted protein, we screened TFPs for hIL-32, a candidate anticancer and anti-immune disease drug^27^. The *hIL32* gene, flanked with linker DNA and a 0.3-kb DNA fragment encoding an N-terminal part of invertase, was prepared by overlap extension PCR and applied to the TFP library as described before. Around 250 transformants were formed on sucrose medium, compared to 3 × 10^4^ transformants on UD medium. Plasmid DNA was recovered from the 30 largest colonies, and the *hIL32* gene was expressed without *SUC2* directed by each TFP to select clones that exhibited high levels of hIL-32 protein secretion. Similar to the case of hIL-2, 60% of the TFPs secreted hIL-32 into the culture supernatant with different efficiencies (Fig. 5b). Repeated screening of TFPs for hIL-32 and nucleotide sequencing of 30 high-producing clones revealed 14 different TFPs, including four common TFPs also identified in the screening for hIL-2 (Table 1). Some TFPs were repeatedly found from the same genes, but with different sizes (TFP5 and TFP14). Finally, TFP10 was selected, and fed-batch fermentation was carried out to confirm the secretory productivity of hIL-32. As a result, over 500 mg/L of hIL-32 was produced from 60 g DCW/L of yeast (Fig. 6).

### Direct application of screened TFPs for the production of other proteins

**Figure 7:**
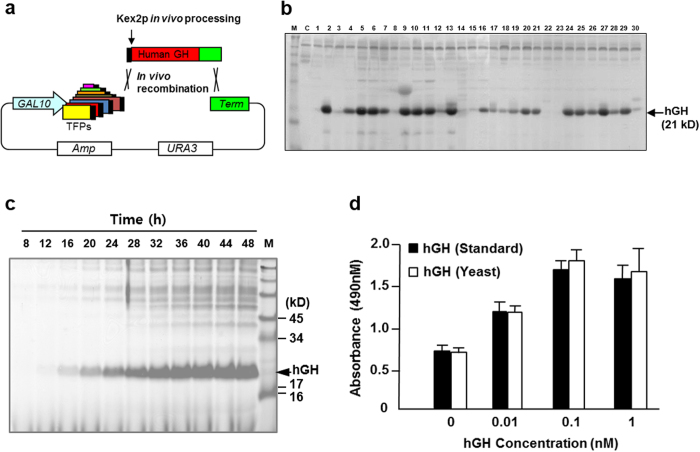
Application of the mini-translational fusion partner (TFP) library for the production of hGH. (**a**) Schematic diagram of direct TFP screening from a mini TFP library. (**b**) SDS-PAGE analysis of hGH expressed by selected translational fusion partners (TFPs). A 0.6-mL aliquot of the culture supernatant was analysed on a 10% Tricine gel after precipitation with acetone. M: standard protein size marker, C: host strain carrying a mock vector, lane 2, 5, 6: TFP5-1, lane 9: TFP4, lane 10, 13: TFP11, lane 11, 27: TFP17-3. (**c**) SDS-PAGE analysis of fed-batch fermentation broth (10 μL) retrieved at the indicated times. (**d**) Bioactivity assay of the purified hGH. The Nb2-lymphoma cell line was cultured in the presence of the indicated amounts of hGH, and cell proliferation was analysed at 490 nm. The protein was revealed by Coomassie staining.

**Figure 8:**
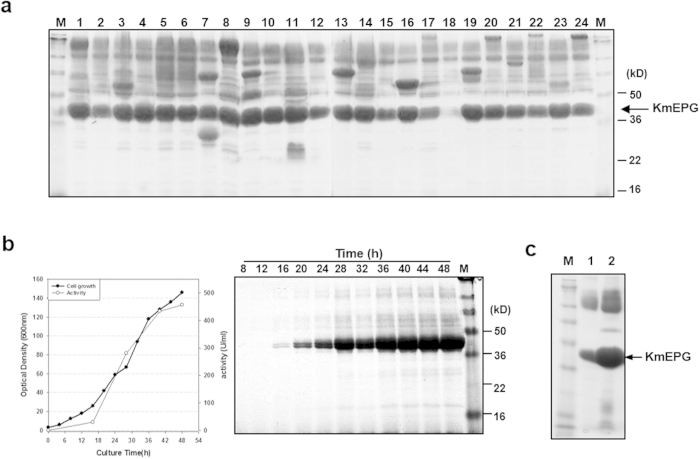
Selection of optimal TFP and production of endopolygalacturonase (KmEPG1) by using the mini-translational fusion partner (TFP) library. (**a**) SDS-PAGE analysis of KmEPG1 expressed by selected TFPs. A 0.6-mL aliquot of the culture supernatant was analysed on a 10% Tricine gel after precipitation with acetone. M: standard protein size marker, lane 1: TFP1-4, lane 2: TFP2, lane 3: TFP3, lane 4: TFP3-1, lane 5: TFP18, lane 6: TFP16, lane 7: TFP4, lane 8: TFP5, lane 9: TFP6, lane 10: TFP7-1, lane 11: TFP8, lane 12: TFP9, lane 13: TFP10, lane 14: TFP11, lane 15: TFP12, lane 16: TFP13, lane 17: TFP14-1, lane 18: TFP15, lane 19: TFP16-3, lane 20: TFP17-3, lane 21: TFP18-1, lane 22: TFP19-1, lane 23: TFP20, lane 24: TFP14. (**b**) Profile of fed-batch fermentation and SDS-PAGE analysis of fermentation broth (10 μL) retrieved at the indicated times. (**c**) Comparison of KmEPG1 secretion level between native secretion signal (lane 1) and TFP3 (lane 2). The protein was revealed by Coomassie staining.

As summarised in Table 1, the 39 TFPs selected by two rarely secreted target proteins were derived from various secretory proteins with different functions and localisations. The lengths of TFPs varied from 51 to 213 aa. Most TFPs were parts of extracellular glycoproteins or membrane proteins with apparent secretion signal sequences. Among them, TFP1-4 and TFP10 were selected as optimal TFPs for the secretion of hIL-2 and hIL-32, respectively. Although the remaining TFPs showed less capacity to induce the secretion of hIL-2 and hIL-32 than the selected TFPs, they were tested for the secretory production of other proteins to demonstrate our initial concept of the different capacities of TFP according to the target proteins. Mini-library vectors containing 39 TFPs were used for the selection of an optimal TFP for the secretion of human growth hormone (hGH). Vectors containing each TFP were constructed as described in the Methods for the simple insertion of target genes between TFPs and the transcriptional terminator. Y2805 was transformed with a mixture of *Swa*I-digested mini-library vectors and the PCR-amplified *hGH* gene flanked with homologous sequences of vector ends to construct a mini-library of transformants through *in vivo* recombination (Fig. 7a). Thirty transformants were selected and cultured to isolate an optimal TFP for the secretion of hGH (Fig. 7b). Most transformants secreted correctly processed hGH (21 kDa) through Kex2p, but with different secretion levels. Yeast strains secreting hGH directed by TFP5-1 (SRL1-59aa), TFP11 (ECM33-55aa), and TFP17-3 (CCW12-125aa) showed higher levels of hGH secretion than others. Although these TFPs were initially isolated for hIL-2 or hIL-32 secretion, they were found to be much more useful for hGH secretion, demonstrating the versatile capabilities of the selected TFPs with regard to the target proteins and the usefulness of the mini-TFP library for the expression of other recombinant proteins. The productivity of hGH was confirmed by fed-batch fermentation of the transformant secreting hGH directed by of TFP5-1. Over 1 g/L of recombinant hGH was secreted from 54 g yeast DCW/L (Fig. 7c). The bioactivity of purified hGH was equivalent to that of standard hGH (Fig. 7d). Another example of TFP selection from the mini-TFP library was performed using an industrial enzyme, pectin-degrading endopolygalacturonase of yeast *Kluyveromyces marxianus* (KmEPG1). A set of 24 TFPs selected from Table 1 were tested individually for the secretion of KmEPG1. Although most of the selected TFPs rendered efficient secretion of KmEPG1 (Fig. 8a), analysis of EPG activity revealed that transformants, including vectors with TFP1-4 (YAR066W-105aa), TFP3 (CIS3-103aa), and TFP16-3 (SCW4-111aa), were higher than others. Selected TFPs for the optimal secretion of KmEPG were quite different from those for hGH, suggesting the different secretion effects of the TFPs on different target proteins. Around 1 g/L of KmEPG1 was secreted from 45 g DCW/L during fed-batch fermentation of a yeast transformant secreting KmEPG1 directed by TFP3 (Fig. 8b). Although KmEPG1 was reported as a poorly secreted protein in recombinant expression using *Pichia pastoris* by the MFα signal sequence of *S. cerevisiae*^28^ and even under its own secretion signal^29^, approximately 10-fold more KmEPG1 secretion was detected using TFP3 as a leader sequence instead of its own secretion signal (Fig. 8c). The results clearly suggested that screening for optimal TFPs could improve the secretion level of each protein and that the TFP selection system developed in this study can be a powerful tool for improving the yields of various recombinant proteins.

## Discussion

Over the past decade, most studies on heterologous protein secretion in yeast have focused on engineering a host strain by inactivation or overexpression of genes related to the secretory pathway or improving the expression vector system and optimising the fermentation process^30^,^31^. Host engineering to manipulate protein folding in the ER and disulfide bond formation has been the main approach to improving the secretion levels of heterologous proteins. In this report, we present an alternative approach for hypersecretion of heterologous proteins by arrangement of optimal TFPs in yeast. In contrast with host engineering, translational fusion of a target protein with a secretory fusion partner can improve target protein expression without affecting the intact structure of the protein or raising the burden of the host strain via overexpression of chaperones and inactivation of factors inhibiting secretion. Unfortunately, however, no reported fusion partners could serve as omnipotent partners for all proteins. In this study, we constructed TFP libraries using the secretion trap technique and developed a high-throughput optimal TFP screening system for rarely secreted target proteins. We used the low-secretion-competent proteins hIL-2 and hIL-32 as model proteins for the selection of TFPs from genomic and truncated cDNA libraries. Thus, we identified an expanded repertoire of fusion partners that will be useful for the selection of optimal fusion partners for numerous target proteins in a matchmaking manner.

About 10% of over 6,000 *S. cerevisiae* ORFs, encoding proteins harbouring a secretion signal peptide and thus passing through the ER, were the source of the TFP candidates for enhancing target protein secretion. Furthermore, several TFPs could be generated using different sized fragments from a single ORF, and all TFPs may have different efficiencies for enhancing protein secretion. Thus, it is conceivable that several thousand TFPs could be generated from hundreds of yeast genes containing different secretion signal sequences. The selected yeast TFPs listed in Table 1 may be a set of powerful TFPs recoverable from *S. cerevisiae*. Most selected TFPs originated from genes encoding cell wall and secreted proteins. Cell wall proteins have been used as fusion partners for incorporation of recombinant proteins into the yeast cell wall; however, most of the studies conducted to date have focused on the anchoring domain^14^,^32^. Two of our selected TFPs containing pre- and pro-peptides (*CIS3* and *HSP150*) have already been reported as fusion partners for the production of xylanase, β-lactamase, rat nerve growth factor receptor, the VP8 rotavirus antigen, lipase, human IL-1β, endoglucanase, glycosyltransferases, rat alpha 2,3-sialyltransferase and laccase^14^,^33^,^34^,^35^,^36^,^37^,^38^,^39^,^40^,^41^. These fusion partners were used to produce target proteins as fusion proteins, while our TFPs were removed at the Golgi complex via an artificially introduced KEX2 cleavage site, resulting intact proteins.

To predict the secondary structures of TFPs affecting the secretion of target proteins, the primary sequences of TFPs were analysed by Kyte & Doolittle hydropathy plots (data not shown). Although conserved structures were not found, except in the signal peptide region, most TFPs contained a stretch of hydrophilic amino acids following the signal peptide. The function of TFPs could be different on a case-by-case basis, and this may play a role in proper folding of target proteins, as described in studies in which the 5′ coding region of a gene influenced translational initiation and gene expression in *E. coli*^42^,^43^. This would change the low-secretion-competent proteins to more permissive proteins to the secretion pathway^15^,^36^.

TFP1, screened from the genomic DNA library as an optimal fusion partner for hIL-2, was surveyed by comparing the secretion enhancing effects of serially deleted TFP1 variants. Deletion of the serine-alanine (SA)-rich domain of TFP1 resulted in loss of secretion-enhancing activity (Fig. 2). The SA-rich region has been hypothesised to function as a membrane-spanning flexible linker in *S. cerevisiae* by simple sequence analysis; however, the role of the SA-rich region in secretory processing is not clear^44^. Although the exact secretion-enhancing mechanism of TFP has not been identified, we confirmed that the signal sequence, N-glycosylation site, and SA-rich region of TFP1 domains were absolutely required to maintain the secretion-enhancing effects of TFP1. This result clearly indicated that the signal peptide is not always sufficient to ensure high-level secretion of heterologous proteins in yeast. For efficient secretion of some proteins, downstream regions of signal sequence in mature proteins may be critical, as in the case of the pro-region of MFα. TFP1 was screened from a genomic DNA library, not from a cDNA library, probably because the transcript level was insufficient to be screened or because the ORF was not functional under the applied growth conditions^45^. Thus, genome-wide TFP selection for certain target proteins may be improved by using both genomic and cDNA libraries to increase the chances of obtaining an optimal TFP.

The selectivity of the TFP trap system is mainly dependent on the secretion competency of a target protein. Low-secretion-competent proteins, such as hIL-2, act as strong secretion blockers that yield a restricted number of TFPs and show higher selectivity than the more permissive protein, hIL-32. Although about 60% of the TFPs screened on sucrose medium for hIL-2 and hIL-32 secretory expression showed detection of each protein on SDS-PAGE (Fig. 5), there were still many transformants showing no bands. Therefore, it is conceivable that the secreted proteins were not sufficiently abundant to be detected, while the fused invertase activity of transformants was sufficient for survival on sucrose medium. Thus, to improve the TFP selectivity of various proteins with different secretion competencies, it may be helpful to develop a less-active invertase by enzyme devolution.

To demonstrate the usefulness of the TFPs identified in this system for improving yields and secretion of biopharmaceutical or bioindustrial recombinant proteins, whose requirement has increased recently^46^, hGH and KmEPG1 were tested. By direct application of cloned TFPs for expression of these proteins, recombinant strains secreting more than 1 g/L of target proteins were easily obtained. TFP libraries can also be applied to secretion of cellulases, lipases, human growth factors, and others. The TFP screening system and its libraries are strong tools for the expression of various therapeutic and industrial proteins that are difficult to produce using conventional protein secretion systems; however, this system can likely not be used for production of all types of proteins. Indeed, we expect that it will be difficult to find optimal TFPs for the high level secretion of peculiar proteins containing complicated structures that do not exist in yeast, such as PDGF, which contains a cysteine knot; membrane proteins, which contain transmembrane domains; and low secretion competent cytosolic proteins in their native organism. Additional expression of chaperones, engineering of secretory pathways, and identification of novel TFP candidates from other organisms may be helpful to fill up a deficiency in the genome-wide TFP trap system^6^. The optimal fusion partner screening system developed in this study could expand the availability of yeast as a platform host strain for the secretory production of many valuable heterologous proteins of pharmaceutical and industrial interest.

## Methods

### Strains and media

*E. coli* DH5α [F^−^ *lac*ZΔM15 *hsd*R17(r-m-) *gyr*A36] was used for general recombinant DNA techniques. The haploid yeasts, *S. cerevisiae* Y2805 (*Mat* α *pep4::HIS3 prb1 can1 his3-200 ura3-52*) and Y2805*Δgal80* (Y2805 *gal80::Tc190*)^47^,^48^,^49^ were used as general hosts for gene expression and genomic or cDNA isolation. The invertase-defective strain Y2805*Δgal80Δsuc2* (Y2805 *gal80::Tc190 suc2::Tc190*) were constructed by targeted gene disruption with the *URA3* pop-out cassette^50^. All yeast transformations were performed using the lithium acetate method^51^. Yeast cells were generally grown on YPD (1% yeast extract, 2% Bacto peptone, and 2% glucose). General selection of yeast transformants was performed on UD (0.67% yeast nitrogen base without amino acid, 2% glucose, 0.5% Bacto casamino acid, and 2% agar) plate medium. The transformed Y2805*Δgal80Δsuc2* secreting the target protein fused to invertase was selected on YPSA (1% yeast extract, 2% Bacto peptone, 2% sucrose, 2 μg/mL antimycin, and 2% agar) for TFP screening. A seed culture for fermentation was incubated in a 1-L Erlenmeyer flask containing 200 mL of UD broth overnight at 30 °C. The cultured seed (200 mL) was inoculated into a 5-L jar fermentor (Kobiotech, Seoul, Korea) containing 1.8 L of medium consisting of 2% glucose, 4% yeast extract, and 1% Bacto peptone. When the Y2805 strain was used as expression host, a feeding medium containing 300 g glucose, 300 g galactose and 150 g yeast extract (per litre) was used after complete exhaustion of glucose, and in case of Y2805*Δgal80* a feeding medium containing 300 g glucose and 150 g yeast extract (per litre) was used. The hourly feeding rate was manually increased from 2 to 10 g/L of carbon source based on cell growth. The fermentation pH value was controlled at 5.5 with 25% NH_3_.

### Plasmid construction

The primers used in this study are summarised in Supplementary Table 1. To construct the plasmid, pYGAP-SUC2, containing two in-frame fused *Sfi*I recognition sites and a Kex2p cleavage site (LDKR) between the secretion signal peptide and the mature invertase gene (*SUC2*), the glyceraldehyde-3-phosphate dehydrogenase (*TDH3)* promoter, secretion signal, and mature *SUC2* gene were amplified separately with the primer sets GAP-F/GAP-R, SIG-F/SIG-R, and INV-F/INV-R, respectively. The amplified fragments were annealed to a single fragment by using overlap extension polymerase chain reaction (PCR) and were cloned into the *Sac*I/*Sal*I site of a yeast-*E. coli* shuttle vector YEGα-HIR525^52^ after *Sac*I/*Sal*I digestion. The pYGAP-HSA-SUC2 plasmid, containing an in-frame fused *HSA* gene between the signal peptide and the *SUC2* sequence, was constructed by subcloning the *HSA* gene amplified from pYHSA5^53^ using HSA-F and HSA-R primers into the *Sfi*I sites of the pYGAP-SUC2 vector. The pYGAP-hIL2-SUC2 plasmid, containing an in-frame fused *hIL2* gene instead of the *HSA* gene, was constructed by subcloning the *hIL2* gene synthesised at Bioneer (Daejeon, Korea) using a public sequence into the *Sfi*I sites of the pYGAP-SUC2 vector.

The YGaINV plasmid, containing *Sfi*I, *Not*I and *Sfi*I sites between *GAL10* promoter and the mature *SUC2* gene, was constructed for the signal sequence trap from cDNA. The mature *SUC2* gene was amplified from pYGAP-SUC2 using the Sfi-INV-F and INV-R primers. Then, an *Eco*RI-*Sal*I digested PCR fragment was cloned into the *Eco*RI-*Sal*I sites of YEGα-HIR525. The *in vivo* recombination vector, YGadV45, containing two *Sfi*I recognition sites, a linker sequence, a *Swa*I recognition site, and a defective *SUC2* (d*SUC2*) construct in sequence, was constructed by subcloning the *dSUC2* gene amplified from YGaINV using the INV45-F and INV-R primers into the *Not*I-*Sal*I sites of YGaINV.

### Screening of optimal TFPs from genomic DNA for secretion of hIL2

Three vectors, each containing *Bam*HI site between *GAL10* promoter and one of three different reading frames of the *hIL2-SUC2* fusion gene were constructed to create the TFP library from genomic DNA. The *GAL10* promoter was amplified from YEGα-HIR525 with the primer set GAL10-F/GAL10-R and the *hIL2-SUC2* gene was amplified from pYGAP-hIL2-SUC2 with three forward primers, i.e., IL-F0, IL-F1, and IL-F2, and a reverse primer, SUC-R, respectively. The three *hIL2-SUC2* gene fragments were annealed to *GAL10* promoter by using overlap extension PCR and subcloned into the *Sac*I/*Sal*I sites of YEGα-HIR525 after *Sac*I/*Sal*I digestion. Genomic DNA from Y2805 was partially digested with *Sau*3AI and fractionated by electrophoresis on 0.9% agarose gels. Approximate 0.5–1-kb DNA fragments were isolated and cloned into the *Bam*HI site of the three vectors, respectively. The genomic DNA library was recovered from 2 × 10^5^ *E. coli* colonies and introduced into *S. cerevisiae* Y2805Δ*gal80*Δ*suc2*. Transformed cells were plated on YPSA medium and incubated at 30 °C for 3–5 days to screen the TFPs.

### Construction of the TFP library from cDNA

Total RNA was isolated from *S. cerevisiae* Y2805 cultivated to the mid-exponential phase in YPD media to construct the cDNA library. Poly (A)^+^ mRNA was purified from total RNA using an Oligotex mRNA kit (Qiagen, Hilden, Germany). Complementary DNA was synthesised from the isolated mRNA by using a SMART cDNA Synthesis kit (Clontech, Mountain View, CA, USA). A custom ASA24N6 primer was used to synthesise the 5′ partial fragments of cDNA instead of the primer included in the SMART kit. First-strand cDNA was used as the PCR template for double-stranded cDNA synthesis with the 5′ PCR primer of the SMART kit and the ASA24 primer. The PCR conditions included 20 cycles of 95 °C for 30 s and 68 °C for 6 min, as recommended by the manufacturer. Amplified cDNA was treated with phenol/chloroform/isoamyl alcohol (25:24:1) and precipitated with two volumes of ethanol and 0.1 volume of 3 M sodium acetate (pH 5.0). The recovered cDNA was digested with *Sfi*I at 50 °C for 2 h and then fractionated using agarose gel electrophoresis. DNA fragments of 0.3–0.8 kb were isolated from the agarose gel using a gel extraction kit (Bioneer). Extracted DNA was ligated with the *Sfi*I-digested YGaINV vector and transformed into *E. coli* DH5α. About 5 × 10^4^ *E. coli* colonies were pooled, and all plasmids containing a randomly primed cDNA library fused to the *SUC2* gene were isolated using a plasmid isolation kit (Bioneer). Y2805Δ*gal80*Δ*suc2* strain was transformed with the cDNA libraries constructed in the YGaINV vector (YGa-cDNA-INV), spread on YPSA media, and incubated at 30 °C for 3–5 days to screen the TFPs. All transformants formed on YPSA media were transferred to UD plates with toothpicks and incubated at 30 °C for 2 days, respectively. TFP library (YGa-TFP-INV) was isolated from the pooled cells and amplified in *E. coli* DH5α. Finally, *Sfi*I-digested TFP fragments were transferred to the same sites of the YGadV45 plasmid for construction of *in vivo* recombination vector, YGa-TFP-dV45.

### Fusion partner trap

Target genes were amplified using the target-specific forward (KR-target-F) and reverse primers (Target-INV-R) from a cloned target gene for in-frame fusion of target proteins with the TFP library constructed in YGadV45 through *in vivo* recombination. Another PCR to amplify the N-terminal part of *SUC2*, which was to be fused to the 3′-end of a target gene, was also carried out using a forward primer (KR-Inv-F) and a reverse primer (Inv300-R) from YGaINV. These two fragments were annealed into a single fragment using overlap extension PCR with the LNK40 and Inv300-R primers. The fused fragment was mixed with *Swa*I-digested TFP library vectors constructed in YGadV45 (YGa-TFP-dV45) and used for *S. cerevisiae* Y2805Δ*gal80*Δ*suc2* transformation. Transformed cells were selected in YPSA media.

### Characterisation of the TFPs and construction of mini-TFP library

To remove the *SUC2* gene of YGaTFP1-hIL2-SUC2 screened from the genomic DNA library, the *GAL10* promoter-TFP1-hIL2 gene was amplified with the GALSac-F and IL2Ter primers. Then, the *Sac*I-*Sal*I-digested PCR fragment was subcloned into the same sites of YEGα-HIR525, and the resulting plasmid was named YGaTFP1-hIL2. To add a Kex2p recognition site (LDKR) between TFP1 and hIL2, the *GAL10* promoter-TFP1 and hIL2 were amplified using GALSac-F/T1KR-R and KRIL-F/IL2Ter primer sets, respectively. Then, two PCR fragments were annealed to a single fragment with overlap extension PCR and cloned into the *Sac*I-*Sal*I sites of YEGα-HIR525, generating YGaTFP1-KR-hIL2. Four TFP1-truncated plasmids were constructed by PCR from YGaTFP1-KR-hIL2 as a template to analyse functional domains of TFP1. Three PCR fragments containing the sequentially truncated *TFP1* gene were amplified using the GALSac-F common forward primer and three different reverse primers, i.e., TFP1-1R, TFP1-2R, and TFP1-3R, digested with *Sac*I-*Xba*I, and cloned into YGaTFP1-KR-hIL2. The resulting plasmids were named YGaTFP1-1-KR-hIL2, YGaTFP1-2-KR-hIL2, and YGaTFP1-3-KR-hIL2, respectively. YGaTFP1-4-KR-hIL2 was constructed by co-inserting a *Bam*HI-*Sal*I-digested PCR fragment amplified using the TFP1-4F and IL2Ter primers from the YGaTFP1-KR-hIL2 plasmid and *Sac*I-*Bam*HI-digested *GAL10* promoter into *Sac*I-*Sal*I sites of YEGα-HIR525.

To construct mini-TFP library, all TFP genes listed in Table 1 were amplified with GAL40 (located in the *GAL10* promoter) and the LNKS-R primer and then cloned between the *GAL10* promoter and *GAL7* terminator of the YEGα-HIR525 vector using an In-Fusion HD cloning kit (Clontech), respectively. To express the *hGH* and *KmEPG1* genes using the mini-TFP library, the mature regions of respective genes were amplified with hGH-F/hGH-R and KmEPG-F/KmEPG-R primer sets, respectively. Sense (F) primers contained 17 nucleotides of the linker sequence and antisense (R) primers contained 20 nucleotides of the *GAL7* terminator sequence for addition of homologous recombination site by second PCR with the LNK40/GT50R primer sets. Recombinant *S. cerevisiae* Y2805Δ*gal80* strains were directly constructed by co-transformation of the *Swa*I-digested TFP vectors and target genes re-amplified with the LNK40/GT50R primer set.

### Protein analyses and purification

Recombinant cells containing a foreign protein expression vector were cultivated in test tube containing 4 mL broth media. Then, 0.6 mL of culture supernatant was mixed with 0.4 mL of cold acetone. After a 2-h incubation at −20 °C, proteins were precipitated by centrifugation for 15 min at 10,000 × *g*. The pellets were freeze-dried and resuspended in 1 × SDS-PAGE sample buffer (Bio-Rad, Hercules, CA, USA) and analysed on 10%–12% Tris-glycine or Tricine gels under denaturing conditions. A 10 μL aliquot of culture supernatant was directly used for SDS-PAGE after mixing with 2 × SDS-PAGE sample buffer and stained with Coomassie blue to analyse the secreted proteins from the fed-batch fermentation. Total intracellular yeast protein was prepared from the cells by post-alkaline extraction^54^. A polyclonal antibody to hIL-2 (R&D Systems Inc., Minneapolis, MN, USA) and an anti-goat IgG alkaline phosphatase conjugate (Sigma Chemical Co., St. Louis, MO, USA) was used for western blot analysis. Secreted hIL-2 and hGH were purified by cation exchange and gel filtration column chromatography. The fermentation broth containing target proteins was filtered with a 0.1-mm Sartoclear filter (Sartorius AG, Goettingen, Germany) and concentrated by ultrafiltration with a 10 K NMWC Quick-stand (GE Healthcare, Piscataway, NJ, USA) using buffer A (20 mM sodium acetate, pH 5.0). The concentrated proteins were loaded onto a SP Sepharose column (2.2 × 10 cm, GE Healthcare) equilibrated with buffer A, and target proteins were eluted with a 0–1 M NaCl linear gradient (buffer B: buffer A + 1 M NaCl). The pooled and concentrated eluents were purified by Superdex 75 (1.6 × 60 cm, GE Healthcare) gel filtration chromatography using elution buffer (20 mM Tris-Cl and 20 mM NaCl, pH 7.4). The concentration of produced proteins were determined by image densitometer using standard proteins.

The bioactivities of hIL-2 and hGH were determined by lymphocyte proliferation assays using the EL-4 and Nb2 cell lines, respectively. Cells were cultured in flat-bottomed 96-well plates and supplemented with various concentrations of recombinant proteins. The proliferation of EL-4 and Nb2 cells was analysed using an Enzyme-Linked Immunosorbent Assay BrdU kit (Roche, Mannheim, Germany) and CellTiter 96 Aqueous Assay kit (Promega, Madison, WI, USA), respectively.

## Additional Information

**How to cite this article**: Bae, J.-H. *et al.* An Efficient Genome-Wide Fusion Partner Screening System for Secretion of Recombinant Proteins in Yeast. *Sci. Rep.* **5**, 12229; doi: 10.1038/srep12229 (2015).

## Supplementary Material

Supplementary Table 1

## Figures and Tables

**Table 1:** List of translational fusion partners (TFPs) selected in this study.

TFP	Standard name	Systematic name	Amino acid	Target	Source	Features^a^	Cellular localization^b^
TFP1-4	—	YAR066W	105	hIL2	Genomic	Pre-SS, N-gly, Ser, Ala-rich, GPI	CM
TFP2	ULI1	YFR026C	117	hIL2	Genomic	Pre-SS, N-gly, TMD	Unknown
TFP3	CIS3	YJL158C	103	hIL2	Genomic	Pre-pro-SS, O-gly, PIR	S, CW
TFP3-1	CIS3	YJL158C	87	hIL2, hIL32	cDNA	Pre-pro-SS, O-gly, PIR	S, CW
TFP4	VOA1	YGR106C	213	hIL2	cDNA	Pre-SS, N-gly, TMD	VM
TFP5	SRL1	YOR247W	51	hIL2	cDNA	Pre-SS, N-gly, O-gly, Ser, Thr-rich	S, CW
TFP5-1	SRL1	YOR247W	59	hIL32	cDNA	Pre-SS, N-gly, O-gly, Ser, Thr-rich	S, CW
TFP5-2	SRL1	YOR247W	81	hIL32	cDNA	Pre-SS, N-gly, O-gly, Ser, Thr-rich	S, CW
TFP5-3	SRL1	YOR247W	112	hIL2	cDNA	Pre-SS, N-gly, O-gly, Ser, Thr-rich	S, CW
TFP6	SIM1	YIL123W	125	hIL2, hIL32	cDNA	Pre-SS, N-gly, O-gly, Ser, Ala-rich,	S
TFP7	OST3	YOR085W	51	hIL2	cDNA	Pre-SS, O-gly	ERM
TFP7-1	OST3	YOR085W	186	hIL32	cDNA	Pre-SS, O-gly	ERM
TFP8	—	YNL190W	64	hIL2	cDNA	Pre-SS, N-gly, internal repeats, CWP	Unknown
TFP9	EMP24	YGL200C	81	hIL2	cDNA	Pre-SS, TMD	ERM
TFP10	HSP150	YJL159W	161	hIL32	cDNA	Pre-pro-SS	S
TFP11	ECM33	YBR078W	55	hIL2	cDNA	Pre-SS, GPI	ERM
TFP11-1	ECM33	YBR078W	196	hIL2	cDNA	Pre-SS, GPI	ERM
TFP12	ATG27	YJL178C	144	hIL2	cDNA	Pre-SS, TMD	ERM
TFP13	UTH1	YKR042W	85	hIL2	cDNA	Pre-SS, SUN family, Ser-rich	MM
TFP14	SED1	YDR077W	157	hIL32	cDNA	Pre-SS, GPI	S, CW
TFP14-1	SED1	YDR077W	182	hIL32	cDNA	Pre-SS, GPI	S, CW
TFP15	BGL2	YGR282C	78	hIL2	cDNA	Pre-SS	S, CW
TFP16	SCW4	YGR279C	53	hIL2	cDNA	Pre-SS, CWP	S, CW
TFP16-1	SCW4	YGR279C	64	hIL2	cDNA	Pre-SS, CWP	S, CW
TFP16-2	SCW4	YGR279C	95	hIL2	cDNA	Pre-SS, CWP	S, CW
TFP16-3	SCW4	YGR279C	111	hIL2, hIL32	cDNA	Pre-SS, CWP	S, CW
TFP17	CCW12	YLR110C	60	hIL2	cDNA	Pre-SS, CWP	S, CW
TFP17-1	CCW12	YLR110C	74	hIL2	cDNA	Pre-SS, CWP	S, CW
TFP17-2	CCW12	YLR110C	112	hIL2	cDNA	Pre-SS, CWP	S, CW
TFP17-3	CCW12	YLR110C	125	hIL2	cDNA	Pre-SS, CWP	S, CW
TFP18	FIT3	YOR383C	67	hIL2	cDNA	Pre-SS, GPI	S, CW
TFP18-1	FIT3	YOR383C	163	hIL32	cDNA	Pre-SS, GPI	S, CW
TFP19	YGP1	YNL160W	67	hIL2	cDNA	Pre-SS, N-gly, CWP	S
TFP19-1	YGP1	YNL160W	125	hIL2	cDNA	Pre-SS, N-gly, CWP	S
TFP20	CCW14	YLR390W-A	102	hIL32	cDNA	Pre-SS, CWP	S, CW
TFP21	PAU23	YLR037C	120	hIL32	cDNA	Pre-SS, TMD, CWP	S
TFP22	SPS100	YHR139C	77	hIL32	cDNA	Pre-SS	Unknown
TFP23	HPF1	YOL155C	110	hIL2, hIL32	cDNA	Pre-SS	S, CW
TFP24	HKR1	YDR420W	70	hIL2	cDNA	Pre-SS, TMD	CM

^a^Pre-SS: pre-secretion signal, Pre-pro-SS: prepro-secretion signal, N-gly: N-glycosylation site, O-gly: O-glycosylation site, GPI: glycosylphosphatidyl inositol anchor protein, PIR: protein internal repeats, CWP: cell wall protein, TMD: transmembrane domain.

^b^CM: cell membrane, S: secreted, CW: cell wall, VM: vacuole membrane, ERM: ER membrane, MM: mitochondrion membrane.
