# Supplementary material for: An Efficient Genome-Wide Fusion Partner Screening System for Secretion of Recombinant Proteins in Yeast
Source: Sci Rep. 2015 Jul 21;5:12229. doi: 10.1038/srep12229 (PMC4508530; doi:10.1038/srep12229)
Supplement: Supplementary Table 1 [file srep12229-s1.pdf]

Supplementary Information

An Efficient Genome-Wide Fusion Partner Screening System for Secretion of Recombinant Proteins in Yeast

Bae et al.

Supplementary Table 1. List of primers used in this study

Primer	Sequence
GAP-F	CGAGCTCGGTACCCAAATTTTCCTATGAC
GAP-R	GAAAGCTTGCAAAAGCATTTTTGAATTCGTTTGTTTATGT
SIG-F	GAATTCAAAAATGCTTTTGCAAGCTTTC
SIG-R	GAGGCGGCCGCACGGCCGTAATGGCCAGCAGATATTTTGGCTG
INV-F	GGCCGTGCGGCCGCCTCGGCCCTAGATAAAAGGTCAATGACAAACGAAAC
INV-R	AGTCGACTTACTATTTTACTTCCCTTACTTG
HSA-F	AGGCCATTACGGCCGTGATGCACACAAGAGTGAG
HSA-R	AGGCCGAGGCGGCCAAGCCTAAGGCAGCTTGAC
Gal10F	TCGAGCTCATCGCTTCGCTGATTAATTAC
GAL10R	GGATCCTTGAATTTTCAAAAATTCTTAC
Sfi-INV-F	AGAATTCAAAAGGCCATTACGGCCGCGGCCGCCTCGGCCCTAG
INV45-F	GCGGCCGCCTCGGCCTCTGCTGGCCTCGCCTTAGATAAAAGATTTAAATGACACCGTATGGGGTA
IL-F0	ATTTTTGAAAATTCAAGGATCCGCACCTACTTCAAGTTCT
IL-F1	ATTTTTGAAAATTCAAGGATCCTGCACCTACTTCAAGTTCT
IL-F2	ATTTTTGAAAATTCAAGGATCCTTGCACCTACTTCAAGTTCT
IL2Ter	AGTCGACTTAAGTTAGTGTTGAGATG
ASA24N6	GCCAGCAGAGGCCGAGGCGGCCAGNNNNNN
ASA24	GCCAGCAGAGGCCGAGGCGGCCAG
KR-Target-F	CTCGCCTTAGATAAAAGA-Target sense sequence
Target-INV-R	CATTGAACGCTTGTCCAA-Target anti-sense sequence
KR-Inv-F	TTGGACAAGCGTTCAATGACAAACGAAACTAGCGATAG
INV300R	GTATCATTGAAAAACCCACTCGTG
GALSac-F	CGAGCTCGGTACCCGGGGATCC
T1KR-R	GAACTTGAAGTAGGTGCCCTTTTATCTAGAGGATCAGATGAGAAGAC
KRIL-F	TCTTCTCATCTGATCCTCTAGATAAAAGGGCACCTACTTCAAGTTC
TFP1-1R	AGAACCGAGAGCGCCGCGAGAG
TFP1-2R	TCTAGAGGTGCTATTGGTGTAAGAG

TFP1-3R	TCTAGAATCACCGCTAATTGTTGTG
ILSpe-F	ACTAGTCTAGATAAAAGGGCACC
TFP1-4-F	GGATCCATGTTCAATCGTTTTAAC
GAL40	GTAAGAATTTTTGAAAATTC
LNKS-R	TGTCACTCCGTTCAAGTCGACATTTAAATCTTTTATCTAAGGCGAG
hGH-F	CTCGCCTTAGATAAAAGATTCCCAACCATTCCCTTA
hGH-R	CACTCCGTTCAAGTCGACCTAGAAGCCACAGCTGC
KmEPG-F	CTCGCCTTAGATAAAAGATCTCCATTGGAAAAGAG
KmEPG-R	CACTCCGTTCAAGTCGACTTAACAGAAGGCTCCGC
LNK40	GGCCGCCTCGGCCTCTGCTGGCCTCGCCTTAGATAAAAGA
GT50R	GTCATTATTAAATATATATATATATATATATTGTCACTCCGTTCAAGTCGAC
